# Uptake and accumulation of Microcystin-LR based on exposure through drinking water: An animal model assessing the human health risk

**DOI:** 10.1038/s41598-018-23312-7

**Published:** 2018-03-20

**Authors:** Brett Greer, Julie P. Meneely, Christopher T. Elliott

**Affiliations:** Institute for Global Food Security, School of Biological Sciences, Queens University Belfast, Stranmillis Road, Belfast, BT9 5AG UK

## Abstract

Harmful Algal Blooms (HABs) in freshwater systems and intensified aquaculture have increased the risk to human health through exposure to cyanotoxins such as microcystin-LR (MC-LR). To understand the uptake and processing of MC-LR in humans, the pig was chosen as an animal model. This was assessed by repeated exposure for 13 weeks of eight animals dosed daily with MC-LR at 0.04 µg/kg bw, repeated with six animals over five weeks at a dose 50 times higher at 2 µg/kg bw. An analytical method was developed for MC-LR in porcine serum and also to analyse levels of *free* MC-LR in harvested porcine tissues, with Lemieux Oxidation employed to determine *bound* MC-LR in these tissues. MC-LR was not detected in the serum of treated animals from either experiment but *free* MC-LR was observed in the large intestine and kidney from two animals from the higher dosed group at levels of 1.4 and 1.9 µg/kg dry weight (dw) respectively. The results indicated 50% of higher dosed animals accumulated *bound* MC-LR in liver tissue, averaging 26.4 µg, approximately 1.1% of the dose administered. These results point to the potential uptake and accumulation of MC-LR in human liver tissue exposed chronically to sub-acute doses.

## Introduction

Harmful Algal Blooms (HABs) are increasing in number and severity in freshwater systems globally with these HABs caused by certain cyanobacterial species growing exponentially and producing secondary metabolites called cyanotoxins. Rises in HABs is in part caused by intensive use of freshwater resources such as lakes and reservoirs as well as pollution of these, resulting in increases in nutrients in the water which can favour cyanobacterial species over ‘true’ algal species^1–3^. Rising global temperatures caused by climate change is predicted to play an increasing role in the future^4^, with higher water temperatures known to promote surface-forming HABs.

Approximately 40 cyanobacterial species are capable of producing cyanotoxins^5,6^ with the most studied being the hepatotoxic microcystins (MCs), of which around 130 different congeners have been characterised^7^. MCs are cyclic heptapeptides, with most structural variations coming from differences in amino acids at positions 2 and 4 within the main cyclic body of the molecule. Microcystin-LR (MC-LR), with amino acids leucine (L) and arginine (R) at positions 2 and 4 respectively^8^, is the most studied and acutely toxic and is listed as one of the most important algal toxins in the United States^9^.

MCs are toxic to humans and animals^8^ with one incident leading to human fatalities in Caruaru, Brazil, when dialysis patients were acutely exposed^10^. Exposure to cyanotoxins occurs when HABs release toxins such as MCs into their surroundings. Often the watercourse is a source of drinking water and 80% of human exposure is thought to result from ingestion of contaminated drinking water^11^, with recreational exposure also documented^12,13^. Further exposure is through consumption of contaminated food, with studies showing the bioaccumulation of cyanotoxins including MCs in freshwater *seafood*^14,15^, with this route more than likely underestimated, especially with rises in aquaculture^16,17^.

Due to its toxicity and based on the No Observable Adverse Effect Level (NOAEL) value of 40 µg/kg body weight in the 13 week drinking water study carried out in mice with MC-LR (Fawell *et al*. 1994), the World Health Organisation (WHO) established a 1 µg/L (free plus cell-bound) guideline value for drinking water and a Tolerable Daily Intake (TDI) of 0.04 µg/kg body weight per day for MC-LR in contaminated *seafood*^11^.

MCs primary target organ is the liver with their main mechanism of action being induction of liver failure by inhibition of protein phosphatases type 1 and 2A (PP1 and PP2A) in hepatocytes^18^. Their transportation and uptake into the liver through the bile acid transport system is mediated through organic anion-transporting polypeptides (OATPS) which are expressed in hepatocytes^19^. However, OATPs are known to be expressed in other organs such as the stomach, small and large intestines, kidney and brain^20^, intimating that MCs could target other organs. There is evidence suggesting MCs are genotoxic due to their ability to damage DNA and promote tumours^21,22^, with them also considered tumour initiators^23^. A correlation has been drawn between high levels of primary liver cancer (PLC) in regions of South East Asia and China and the drinking of surface water where cyanotoxins are reported to be abundant as opposed to well water^21^, and in 2010 MC-LR was classified as being possibly carcinogenic to humans^24^. Recent studies suggest that MC-LR may promote the migration and invasion of colorectal cancer and it has been implicated in renal-function impairment^25,26^. MC-LR has also been shown to cause apoptosis and changes to the permeability of the mitochondrial membrane in mice^27^ and can cause suppression of haematopoiesis function in mice leading to normocytic anaemia through prolonged exposure^28^. To date, the only human study looking at daily exposure and subsequent health effects of chronic exposure to MCs was conducted by Chen *et al*. (2009). They identified MCs in the serum of fishermen, most likely from exposure to ingesting MCs from the water and fish they consumed, and showed certain enzymes linked to liver function were elevated in their serum, indicating hepatocellular damage^29^.

As discussed previously, MCs can bioaccumulate in tissues and do this as *free* or *bound* (*masked*) through their conjugation to PP1 and PP2A in certain tissues. Binding of MCs to protein phosphatases is irreversible and they cannot be detected by conventional extraction techniques which deal solely with extraction of *free* MCs. Instead, use of Lemieux oxidation, known to produce 2-methyl-3-methoxy-4-phenylbutyric acid (MMPB) through oxidation of the Adda side chain common to every MC congener^30^, allows identification of *bound* MC.

**Figure 1:**
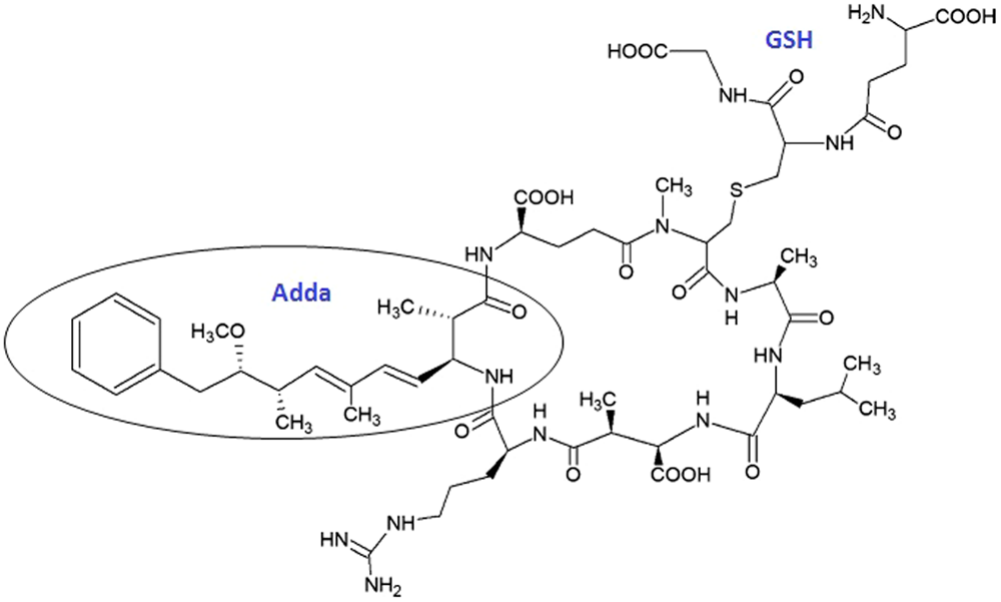
Structure of the microcystin-LR-glutathione conjugate (MC-LR-GSH) showing conjugation between the thiol group of GSH to the unsaturated carbonyl moiety Mdha of MC-LR. The figure also shows the Adda side-chain, common to all MC congeners and responsible for the molecules toxicity.

In addition to detection of *free* and *bound* MCs, metabolites formed during detoxification of MCs in the liver may also be analysed. The major pathway in depuration of MCs in mammals and aquatic organisms is formation of conjugates such as that formed with glutathione (GSH), MC-LR-GSH (Fig. 1)^31–33^. This analyte is known to be formed in the liver as part of the detoxification process and has also been detected in the kidney of fish, although at lower levels than in liver^31,33,34^.

Although various epidemiological studies have been conducted on chronic exposure to MCs through ingestion of contaminated drinking water and their link to PLC^35–42^, there have yet to be any conclusive studies showing the effects of chronic exposure to MCs apart from that conducted by Chen *et al*. (2009) which indicated hepatocellular damage. To accurately assess the damage caused by chronic exposure to MCs in humans, toxicological studies are required, however this cannot be the case. In order to assess the human risk to cyanotoxins such as MC-LR through consumption of drinking water contaminated with these requires both epidemiological and toxicological studies, with the most detailed research on cyanotoxin toxicology to date having been evaluated in rodents^43^. To extrapolate from mouse to human toxicity, particularly through the use of intraperitoneal injection, requires assumptions that may not be strictly accurate, as are those studies conducted in other vertebrates such as sheep, cattle and rats. In order to obtain reliable data for the risk to humans from consumption of contaminated water, we need to study the oral ingestion of these toxins, with this previously conducted in mice to derive the safety guideline concentrations. However, it has been indicated that the mouse has a higher sensitivity to the effects of such toxins^43^. Therefore, in the experiments conducted herein, the pig has been used as the experimental model due to its gastrointestinal tract, liver and kidney functions being similar to humans. Furthermore, the body weight of mature pigs as well as their metabolic rates is similar to humans^44,45^.

The aim of this research was to use the pig as a model to study the effects of subchronic exposure to MC-LR through oral gavage over a period of time, carried out at both the TDI of 0.04 µg/kg bw per day and at 2 µg/kg bw per day, 50 times the TDI. The objectives of this were to: a) develop and validate a UPLC-MS/MS method for analysis of *free* MC-LR in porcine serum; b) analyse the level of *free* and *bound* MC-LR in six porcine tissues using UPLC-MS/MS; c) screen for the depuration product, MC-LR-GSH in specific tissues and d) assess the uptake and processing of MC-LR in serum and various tissues.

## Results

### Method Validation of MC-LR in porcine serum

The method was fully validated to EC Directive 2002/657 for detection and quantification of MC-LR (See Supplementary information online). As the MC-LR used in the validation was not a Certified Reference Material (CRM) and as the MC-LR may not act as it does in a real sample, EC directive 2002/657 states that at the spiking level used of 5 ppb (5 ng/ml serum), the minimum trueness has to be in the range −30% to +10%. Taking this into account, the resulting validation showed the method to be fit for purpose, showing the trueness to be within the range stipulated with a value of −0.8%. The CC_α_ and CC_β_ values were based on the less intense qualifying ion (*q*) and with an injection volume of 2.5 µL, the mean on-column CC_α_ and CC_β_ values are 0.075 and 0.1 pg (ppt) respectively (Table 1).

**Table 1:** Validation data for *free* MC-LR in porcine serum. *Absolute recovery expressed as extraction efficiency when spiked at 1.25 ng toxin per 250 µL serum, equating to 5 ng/mL.

	Fortified Porcine Serum 5 ng/mL
Intra-day RSD (%) (n = 7)	3.8
Inter-day RSD (%) (n = 20)	3.8
Average conc. (repeatability) (ng/mL)	5.2
Average conc. (reproducibility) (ng/mL)	5.2
Absolute recovery (%)*	85.1
CC_α_ (ng/mL)	0.03
CC_β_ (ng/mL)	0.04

### Sample analysis: Experiment 1

#### Porcine serum: free MC-LR

Analysis of the pre-bleed serum (day 7) from the eight control and eight treated animals (n = 16) was found to be free from MC-LR. Extraction and analysis of the serum from all time points for the eight treated animals (n = 104) was subsequently performed, the results of which showed that no MC-LR was found at the detection limit of the method of 0.03 ng/mL serum.

#### Porcine liver tissue: free MC-LR and MC-LR-GSH

With no MC-LR detected in the serum from any of the eight treated animals, it was decided to also test the liver, kidney, large and small intestine tissues from the eight treated animals for *free* MC-LR in experiment (1). Initially, a sample of liver, kidney, large and small intestine tissues from the eight control animals were screened for *free* MC-LR and found to be absent of the toxin as expected. The liver, kidney, large and small intestine tissues from the eight treated animals were then extracted and analysed, the results of which showed that none of the tissues contained any trace of *free* MC-LR.

The liver and kidney tissues from the eight treated animals were then screened for the depuration product, MC-LR-GSH, with both of these tissues negative for MC-LR-GSH.

#### Porcine tissue: bound MC-LR

Liver tissue from each of the eight treated animals was screened for *bound* MC-LR (MMPB) using Lemieux Oxidation, the results of which showed that the livers from the eight treated animals were negative for MMPB and therefore any *bound* MC-LR.

### Sample analysis: Experiment 2

#### Porcine serum: free MC-LR

Extraction and analysis of serum for each time point from the six treated animals, including pre-bleeds from the control and treated animals (n = 42), was performed and assayed alongside a seven point extracted matrix matched calibration curve in the range 0.2–100 ng/mL for determination of any MC-LR detected. As with experiment (1), all pre-bleed serum samples from the control and treated animals were negative for MC-LR. More importantly, serum from each time point from the six treated animals proved to be negative for *free* MC-LR at the LOD of the method.

With MC-LR and its congeners, as well as any potential metabolites of MC-LR having in common the shared Adda group ((2S, 3S, 8S, 9S)-3-amino-9-methoxy-2, 6, 8-trimethyl-10-phenyldeca-4(E), 6(E)-dienoic acid) (Fig. 1)^8^, serum samples were analysed for parent ions of Adda, with this fragment having an m/z of 135. This was optimised using the MC-LR standard and indicated a peak at the same retention time as when analysed in MRM mode, with the retention window extended to allow identification of any potential MC-LR metabolites.

A selection of serum sample time points from experiment (1) were chosen (n = 32), with; t = 14, t = 42, t = 70 and t = 98 correlating to the start, one, two and three months of treatment for each animal, as well as serum from all time points (n = 36) for each animal from experiment (2). These all proved negative for any peaks in the retention window scanned, with no peak seen at the retention time of MC-LR, further confirming the samples to be negative for *free* MC-LR and not indicating the presence of any MC-LR metabolites.

#### Porcine tissue: free MC-LR and MC-LR-GSH

**Figure 2:**
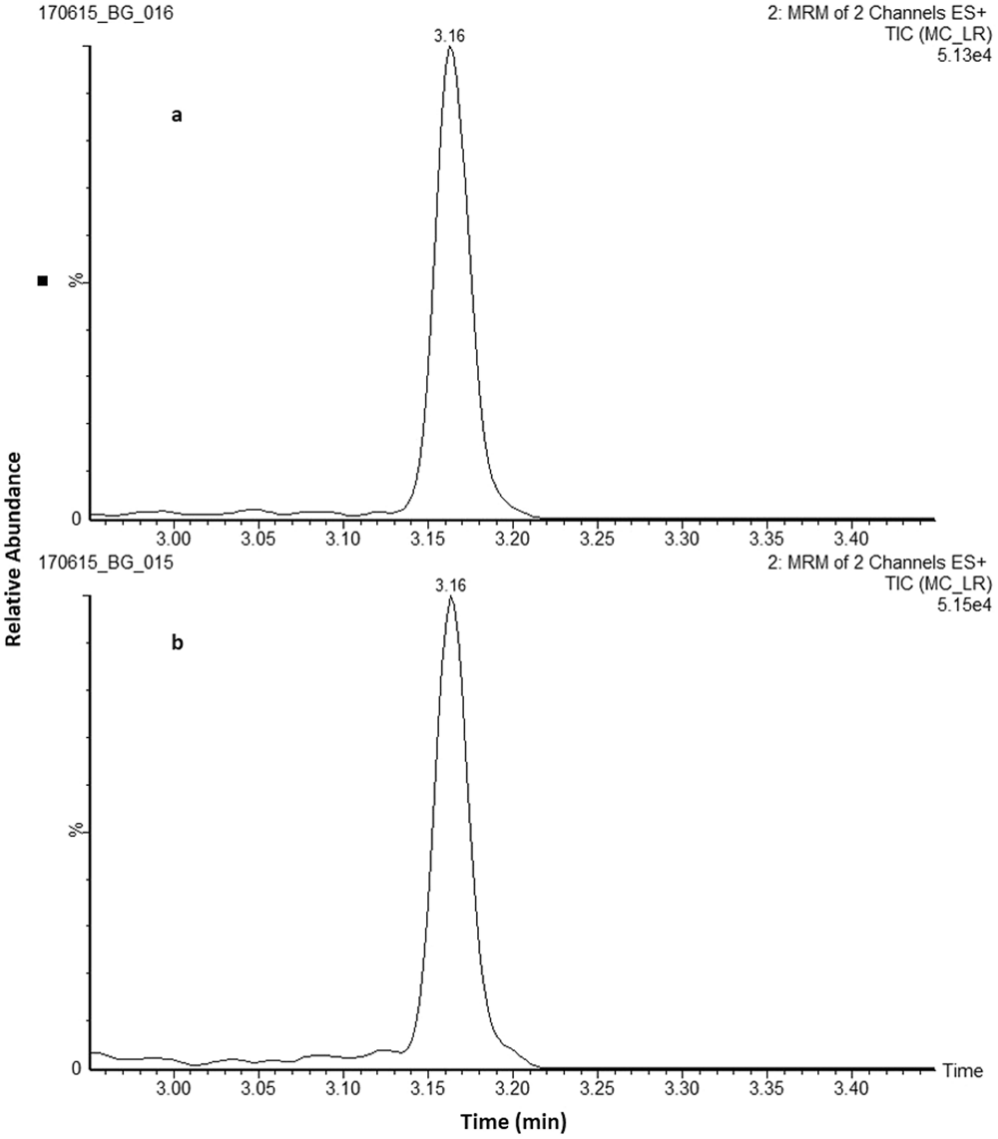
Ultra-high performance liquid chromatography-tandem mass spectrometry analysis of *free* MC-LR in animal tissues. Chromatograms showing samples positive for *free* MC-LR in (**a**) large intestine of animal T5 and (**b**) kidney of animal T1. (MRM, multiple reaction monitoring).

All tissues harvested from the treated animals in Experiment (2) were tested for *free* MC-LR which included; liver, kidney, small and large intestine, spleen and brain. Tissues from the six treated animals were extracted and analysed (n = 36), the results of which showed two samples with measureable amounts of MC-LR, one in the kidney from treated animal 1 (T1) at 1.9 ng/g dw and one in the large intestine from treated animal 5 (T5) at 1.4 ng/g dw, with the chromatograms shown in Fig. 2. All other tissue samples analysed were found to be free of MC-LR residues. This is the first report of MC-LR in the tissues of pigs fed MC-LR through oral gavage and supports the findings of other studies, albeit in fish living in freshwater bodies with HABs, which indicated MC-LR in organs other than in the liver^46–48^.

Liver and kidney tissues from the six treated animals were also screened for the depuration product, MC-LR-GSH (See Supplementary information online). The results of this analysis indicated that four (66%) of the treated livers contained MC-LR-GSH, whereas only two (33%) of the kidneys from the six treated animals contained this compound. As this work was qualitative only, no concentrations could be assigned, however the levels detected in the liver appeared higher than those in the kidney based on the raw peak area of the quantifying ion, *Q*.

#### Porcine tissue: bound MC-LR

**Figure 3:**
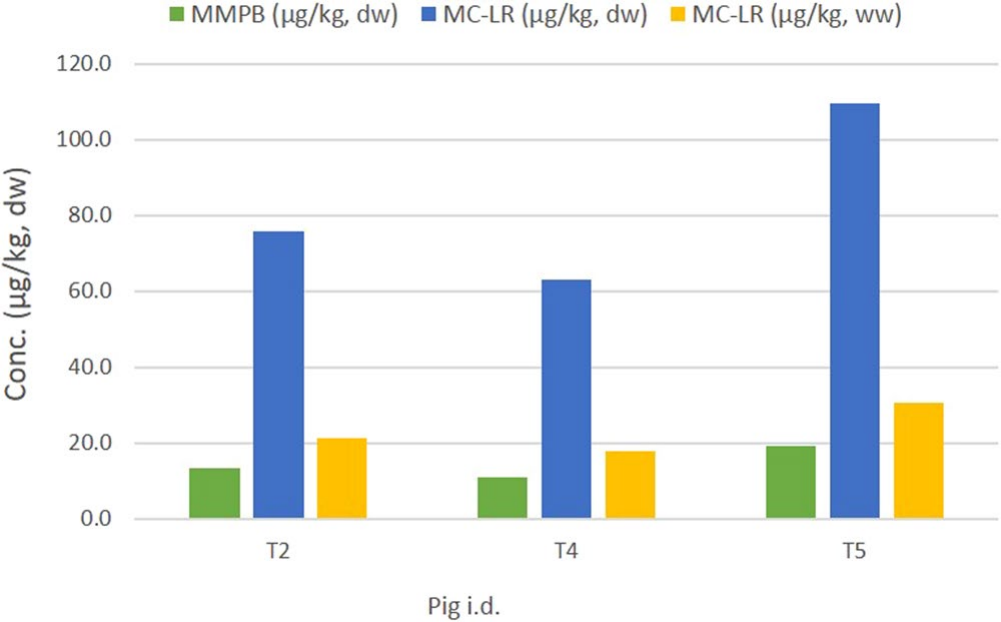
Results of the MMPB detected in the livers from treated pigs from experiment 2 (high dose). Graph shows the amount of MMPB detected, the conversion to molar equivalents of MC-LR and the final estimated value after converting to wet weight.  Concentration of MMPB detected in porcine liver tissue using Lemieux Oxidation.  Molar conversion of MMPB to MC-LR and corrected for loses due to incomplete oxidation based on the oxidation conversion efficiency found on the day of extraction and analysis.  Conversion of concentration detected to wet weight (ww) from dry weight (dw) using a conversion factor of 0.28 based on porcine liver containing approximately 72% water.

Porcine tissues from the six treated animals were initially screened for *bound* MC-LR (MMPB) using Lemieux Oxidation, with MMPB and therefore MC-LR identified in the livers of three treated animals: T2, T4 and T5 (Fig. 3). These were subsequently quantified with the concentrations of MMPB detected ranging from 11.0 to 19.1 ng/g dw, and an average estimated value of 14.4 ng/g dw. On converting the amount of MMPB observed to molar equivalents of MC-LR, and taking into the account the oxidation conversion efficiency of 83% calculated on the day of extraction, the estimated level of MC-LR detected in the livers ranged from 52.4 to 90.9 ng/g dw. Accounting for losses due to incomplete oxidative conversion and calculating back to molar equivalents of MC-LR, the levels detected in the livers ranged from 63.1 to 109.5 ng/g dw.

In must be noted that the water content of porcine liver is approximately 72% (http://slism.com/calorie/111166/) and therefore to achieve a more accurate result for the levels detected, a factor of 0.28 is used to convert from dry weight (dw) to wet weight (ww). On doing so, the estimated levels detected were 17.7 to 30.7 ng/g ww with an average estimated value of 23.2 ng/g ww. Taking the final weight of each pig positive for *bound* MC-LR and assuming the liver weighs approximately 2% of the total bodyweight^49^, the estimated amount of MC-LR which accumulated in each liver equated to 23.1, 21.9 and 34.1 µg for pigs T2, T4 and T5 respectively. With the total dose administered to each animal over the study period known, the percentage uptake and accumulation of MC-LR by each liver can be calculated. This showed the percentage uptake of MC-LR by treated animals T2, T4 and T5 to be 1.1, 0.8 and 1.5% respectively (Table 2), with the average uptake being 1.1% of the total dose administered.

**Table 2:** Total dose administered to the three treated animals positive for MC-LR from experiment 2. Table indicates the levels detected in each liver and the resulting uptake of MC-LR expressed as a percentage of the dose administered.

Pig	Dose (µg)	Pig weight^1^ (kg)	Liver weight^2^ (kg)	MC-LR conc. (µg/kg ww)	MC-LR present^3^ (µg)	Uptake (%)
T2	2191.0	54.5	1.09	21.2	23.1	1.1
T4	2667.0	62.0	1.24	17.7	21.9	0.8
T5	2240.0	55.5	1.11	30.7	34.1	1.5

^1^Weight of pig at end of experiment.

^2^Liver weight calculated as being ~2% of the total bodyweight^49^.

^3^Estimated level of toxin present based on amount of MMPB detected and based on weight of liver at end of experiment.

## Discussion

A UPLC-MS/MS method was developed and validated for the extraction and analysis of *free* MC-LR in porcine serum, showing a method detection limit (CC_α_) of 0.03 ng/mL. The detection limit of the method is an improvement to that used by Chen *et al*. (2009), being over 100 times more sensitive, and with their study requiring in the region of 5 mL of serum, compared to only 250 µL used in the present study, this is an important improvement in the methodology. A further UPLC-MS/MS method was developed for the extraction and analysis of *free* MC-LR across six porcine tissues to allow a detailed study of toxin distribution to take place.

In experiment (1), eight treated animals were dosed with MC-LR daily for 98 days at the WHO TDI value of 0.04 µg/kg bw^50^. No evidence of MC-LR being present at or above the detection limit of the method (0.03 ng/mL serum) was found in the serum or in any of the tissues tested. The study was then repeated over a shorter time scale (35 days) with fewer animals (n = 6) due the costs associated with the toxin, however, this time they were dosed with MC-LR daily at a level of 2 µg/kg bw, 50 times above the WHO TDI. Again, no evidence of MC-LR in the serum of any of the treated animals was found.

Looking at the study by Tencalla *et al*. (1997) in which rainbow trout were dosed with MC-LR also via oral gavage, they estimated the uptake of MC-LR into blood to be approximately 4.4% of the dose administered of 5700 µg MC-LR/kg bw^51^. Based on the findings reported by Tencalla *et al*. and taking the average bodyweight of the six animals in the final week of dosing in experiment (2) of our study, the average dose administered to the six animals equates to 96 µg of MC-LR per day for the last week. Before being euthanized, the average weight of the six animals was 58 kg, with the animals therefore having an approximate blood volume of 3.77 L (65 mL/kg bw) and an average estimated MC-LR concentration of 25.5 ng/mL in their blood. Based on this concentration, and with the Tencalla study seeing 4.4% of the oral dose administered in the blood of the rainbow trout, an approximate concentration of 1 ng/mL should have been present in the serum of the animals from our study. With the LOD (CC_α_) of our method being 0.03 ng/mL, this indicates that MC-LR should have been detectable in the serum, however as reported, no *free* MC-LR was detected in the serum from any of the treated animals. This may however be due to the dose administered in our experiment being significantly lower, approximately 3 000 times less, than that given to the rainbow trout by Tencalla *et al*. as well as it being a different species^51^.

A study conducted by Chen *et al*. on fishermen living beside and subsisting on a lake with HABs showed MCs (-RR, -YR, and -LR) in the serum of the fishermen, with the proportions of the MC congeners identified in their serum matching those found in the fish they were consuming, rather than from the water^29^. This appears to imply that consumption of contaminated *seafood* as opposed to ingestion of contaminated drinking water as being the main route of uptake for these toxins^16^. The majority (77%) of these fishermen lived on the lake for more than 10 years, with their exposure estimated around 2.2–3.9 µg MC-LR equivalents daily, making this chronic long term exposure as opposed to the shorter time frame used in our experiment.

Another factor to consider is that MC-LR appears to be absorbed from the gastrointestinal tract into blood rapidly and is then quickly transported to the liver, with the liver indicating the presence of MC-LR after 1 h and with maximum levels at 3 h after exposure, with a half-life in the blood of 3.3 h^51^. In another experiment conducted by Robinson *et al*. (1991) using radiolabelled MC-LR to track its distribution in mice, levels of radiolabelled MC-LR peaked in the liver after only 30 min^52^, although in this case the mice were injected intravenously *via* the tail vein rather than by oral gavage. Nevertheless, it appears that transport through the intestinal tract into the bloodstream and to the liver occurs rapidly, indicating the possibility that a high proportion of the MC-LR administered could have been cleared from the blood by the time it was collected from the pigs. Also, with the toxin administered through oral gavage in water may be more readily available, which may aid its transport to and subsequent clearance from the bloodstream, whereas being present in food may slow this process down as the toxin has to be released from the matrix it is present in before being processed.

Furthermore, the metabolism of MC-LR in the human gastrointestinal tract has not been fully investigated. A report by the WHO in 1999 indicated no evidence of hydrolysis of MCs by peptidases in the stomach, with a significant amount of what had been ingested being absorbed^53^. However, as MC-LR is a cyclic heptapeptide, there may be the potential for proteolytic degradation of the toxin as it is processed through the gastrointestinal tract, with a study by Moreno and co-workers (2004) demonstrating degradation of several MCs including MC-LR in the gastric phase of digestion *in vitro*^54^. The transit of MC-LR through the intestinal tract was confirmed by the presence of *free* MC-LR at a level of 1.4 ng/g dw (0.29 ng/g ww) in the large intestine of treated animal T5 in experiment (2) of our study. The identification of MC-LR in the large intestine indicates it may accumulate in this tissue, with other studies in fish showing higher levels of MCs in the large intestine than in other tissues in certain species, which may inhibit its transport to other organs such as the liver^48^.

In the second experiment (high dose), with the animals dosed at 50 times above the WHO TDI at 2 µg MC-LR/kg bw per day, all tissues harvested were analysed for both *free* MC-LR and total MC-LR (*free* plus *bound*). The results of these analyses failed to show the presence of *free* MC-LR, apart from the kidney of treated animal 1 (T1) and large intestine from treated animal 5 (T5), with levels detected of 1.9 and 1.4 ng/g dw, or 0.4 and 0.29 ng/g ww respectively.

Analysis of the tissues for *bound* MC-LR revealed 50% of livers from the treated animals in the higher dosed experiment were positive for *bound* MC-LR, with estimated levels of 23.1, 21.9 and 34.1 µg for pigs T2, T4 and T5 respectively (Table 2), averaging 26.4 ± 5.5 µg of MC-LR. These results are the first reporting of *bound* MC-LR in pigs, confirming the uptake and binding of this toxin to proteins in the liver after exposure. Based on the average total dose administered to pigs T2, T4 and T5 of 2 366 µg MC-LR, and indicating an average percentage uptake of 1.1%, this study shows approximately 50% less uptake than that reported by Tencalla *et al*. (1997), with their study indicating 1.7% uptake of MC-LR into the liver when administered via oral gavage, albeit in rainbow trout.

One of the major depuration products in detoxifying MC-LR is MC-LR-GSH, with Kondo *et al*. (1996) identifying its presence in mice and rat livers, and Pflugmacher *et al*. (1998) indicating its formation in the liver as the first stage in the detoxification of MCs^32,33^. MC-LR-GSH is then further biotransformed to a cysteine conjugate, MC-LR-Cys, detected in the kidneys, presumably for excretion via the urine^55^. With this in mind, liver and kidney tissues from the treated animals of both experiments were screened for MC-LR-GSH, a qualitative analysis similar to previous studies^32,33,55^. The results demonstrated the presence of MC-LR-GSH in approximately 66% of the livers and 33% of the kidneys in animals from experiment (2) only. The levels detected in liver were higher than in the kidney (based on raw peak area), with these results similar to other published studies^34,56^.

Furthermore, Falconer *et al*. (1994) conducted an experiment in pigs whereby the animals were dosed orally at concentrations of 1 312, 796 and 280 µg/kg bw, 656, 398 and 140 times respectively higher than that used in experiment (2) of our study. They did not analyse serum or liver tissue for MC-LR (*free* or *bound*), but the results of plasma liver enzyme analyses indicated liver injury. These results support the findings of this study where *bound* MC-LR was detected in liver tissue.

The two pig studies as an animal model for humans indicates MC-LR may be processed from the blood rapidly due to none being detected in serum from any of the treated animals. Some is potentially metabolised as it passes through the gastrointestinal tract and so less may be readily available for uptake into the bloodstream, with this study showing *free* MC-LR in the large intestine, with *free* MC-LR also detected in the kidney. The major finding of this study is of *bound* MC-LR in the livers, with analysis of liver tissue revealing 50% of pigs from the higher dose experiment accumulated MC-LR.

Overall, these findings indicate uptake and accumulation of MC-LR in the liver through sublethal, chronic exposure, and with the pig being a suitable animal model for humans, indicates the possible accumulation in human livers exposed in a similar manner. Further experiments need to be conducted to further probe the link between chronic sub-acute exposure and long term health effects such as PLC.

## Materials and Methods

### Animal Experiment

#### Animal Study 1

The animal studies were performed under licence (PPL 2755 - Chemical contaminants in food producing animals) issued by the Department of Health, Social Services and Public Safety in Northern Ireland (NI) in accordance with the Animal (Scientific Procedures) Act (1986), and under the approval of the Agri-Food and Biosciences Institute (AFBI) Animal Welfare and Ethical Review Body (AWERB) and associated EU Directive 2010/63/EU for animal experiments. The study was performed on behalf of Queen’s University Belfast (QUB) by the Veterinary Sciences Division, Agri-Food and Biosciences Institute, Belfast, Northern Ireland. Sixteen pigs, eight control and eight treated (a cross of Large White and Landrace breeds, PIC 337 breed), each identified by an ear tag were used in the study.

#### Housing

During the study, pigs were housed in individual pens in sight of the other pigs. The individual pens were made of chain link; sawdust/wood shavings prevented the animals from slipping and provided a substance in which the pigs could root. In addition chains were supplied as toys for the animals. The pens met the requirements of EC/2010/63 Directive regarding animal welfare.

#### Diet

Pigs were fed once a day with all groups supplied with the same quantity of food/day, determined by the body weight of the control group, with the daily food intake of each pig recorded throughout the study. The diet was based on the breeder’s recommendations and free access to water was provided by an automated watering system.

#### Treatment

Following the acclimatization period of seven days, eight healthy pigs were selected randomly for the control group and the remaining eight healthy pigs assigned to the treatment group. Before treatment, animals were examined by a veterinarian to confirm clinical health and then weighed, with the weight and identification of animals in each group recorded. Over the study period (13 weeks), each animal from the treatment group received a low dose of MC-LR daily by oral gavage. The dose administered was 0.04 µg toxin/kg body weight, the provisional World Health Organization Tolerable Daily Intake (WHO TDI), based on the NOAEL derived from the animal experiment conducted by Fawell *et al*. (1999), with the average dose for the eight treated animals outlined in Table 3. Briefly, a 1 mg/mL solution of MC-LR was prepared by the addition of 250 µL of ethanol followed by 750 µL water. The appropriate dilutions were then performed depending on the weight of the animal to ensure the correct dose was administered in 5 mL of water.

**Table 3:** Average weight of the eight treated animals for each week of experiment 1 (low dose). Table indicates the average dose administered daily and weekly based on this weight and the average overall dose administered over the course of experiment (1), dosed at 0.04 µg/kg bodyweight (bw).

	Wk 1	Wk 2	Wk 3	Wk 4	Wk 5	Wk 6	Wk 7	Wk 8	Wk 9	Wk 10	Wk 11	Wk 12	End*
Average weight of pig (kg)	20	28	36	44	52	60	68	76	84	92	100	108	116
Average MC-LR Dose/day (µg)	0.8	1.1	1.4	1.8	2.1	2.4	2.7	3.0	3.4	3.7	4.0	4.3	n/a
Total Dose per week (µg)	5.6	7.8	10.1	12.3	14.6	16.8	19.0	21.3	23.5	25.8	28.0	30.2	n/a

Average Total Dose = 215.0 µg. ^*^Average weight of pigs at the end of the experiments before being euthanized.

Serum was collected weekly, starting at day -7 from treated and control animals as the pre-bleeds. Serum samples were collected using a Vacutainer system with all samples processed as quickly as possible and the serum stored at −80 °C prior to use. At day 97 (approximately 13 weeks) treated and control animals were euthanized by intravenous barbiturate and the pigs exsanguinated by jugular severance. Liver, kidney, spleen, large and small intestines and brain samples were collected for MC-LR toxin content and stored at −20 °C. All samples were labelled with the date and time of sampling, the animal identification and whether it was from the treated or control group.

#### Animal Study 2

This study was performed as described previously using twelve male Duroc pigs, six control and six treated pigs. Housing, diet and treatment were as described previously with the following modifications:

Animals were subjected to a fourteen-day acclimatization period prior to assignment to control or treated groups. Over the study period, each animal from the treatment group received a daily dose of 2 µg toxin/kg body weight MC-LR by oral gavage, 50 times the provisional WHO TDI, with the average dose for the six treated animals outlined in Table 4

**Table 4:** Average weight of the six treated pigs for each week of experiment 2 (high dose). Table indicates the average dose administered daily and weekly based on this weight and the average overall dose administered over the course of experiment (2), dosed at 2 µg/kg bodyweight (bw).

	Week 1	Week 2	Week 3	Week 4	Week 5	End*
Average pig weight (kg)	22	27	35	43	48	58
Average MC-LR dose/day (µg)	44.3	53.3	69.5	85.5	95.7	n/a
Average dose/week (µg)	310.1	373.1	486.5	598.5	669.9	n/a

Average Total Dose = 2438.1 µg. ^*^Average weight of pigs at the end of the experiments before being euthanized.

, with the MC-LR for dosing prepared as described above. At day 40 (approximately 5 weeks) treated and control animals were euthanized. Sample collection and storage was as outlined for experiment 1.

### Materials

Microcystin-LR (MC-LR) and nodularin (NOD) standards were purchased from Enzo Life Sciences (UK) Ltd and 2-Methoxy-4-phenylbutyric acid (MMPB) was purchased from Wako Pure Chemical Industries Ltd (Japan). All other chemicals used were purchased from Sigma Aldrich, UK, bar the OASIS HLB and OASIS PRiME HLB solid phase extraction (SPE) cartridges (60 mg, 3 cc) which were purchased from Waters, Ireland. The water used was supplied from an in-house 18 MΏ Millipore water system, Millipore (UK) Ltd.

Standards of MC-LR, NOD (IS) and MMPB used for method development, validation and sample analysis were prepared as outlined in the Supplementary Information online.

### Extraction of *free* MC-LR from porcine serum

Serum samples (see Supplementary Information online) (250 µL) were spiked with 15 µL of the NOD (IS) at 0.5 µg/mL and extracted by addition of 1.8 mL 80% aqueous methanol (*v/v*) (0.5% acetic acid *v/v*), vortex mixed briefly and sonicated for 45 min before centrifugation at 16 000 g for 15 minutes. The supernatant was removed and diluted approximately five-fold by addition of water to reduce the methanol content to 15% (*v/v*) for loading onto SPE cartridges. Samples were loaded onto the OASIS HLB cartridges and allowed to drip through under gravity, after which cartridges were washed with water and 20% aqueous methanol (*v/v*). Bound toxin was eluted into clean glass tubes with 2 × 1.5 mL 80% aqueous methanol (*v/v*) containing 0.1% trifluoroacetic acid (*v/v*). The resulting eluents were evaporated to dryness under a gentle stream of nitrogen using a turbovap at 50 °C. Dried samples were reconstituted in 50 µL 80% aqueous methanol (*v/v*) and transferred to a micro vial for analysis.

### Extraction of *free* MC-LR and MC-LR-GSH from porcine tissues

Tissue samples (see Supplementary Information online) (100 mg) were spiked with 10 μL of the NOD (IS) at 0.5 μg/mL and extracted by addition of 1.5 mL of 80% aqueous MeOH (*v/v*) (0.5% acetic acid *v/v*), vortex mixed briefly and sonicated at 35–40 °C for 45 min before centrifugation at 16 000 g for 15 min. Clean-up was performed as described above with the samples reconstituted in 200 µL 80% aqueous methanol (*v/v*) and transferred to micro vials for analysis.

### Lemieux oxidation and extraction of MMPB

Porcine tissue samples (50 mg) were weighed into 50 mL round bottomed flasks with the conversion and subsequent enrichment of MMPB carried out as outlined in ‘*Analysis of conjugated MC-LR in porcine tissue’* (see Supplementary Information online). Samples were initially screened for MMPB, with any samples found to contain toxin quantified by use of a six point extracted matrix matched calibration curve in the range 0.010–0.25 µg/g dw using pooled control tissue of the relevant tissue, with calibrant levels achieved by spiking with the MMPB standard at a concentration of either 0.5 or 0.05 µg/mL after pH adjustment.

### Ultra-performance liquid chromatography tandem mass spectrometry (UPLC- MS/MS)

#### Analysis of free MC-LR in porcine serum

Analysis was performed using an ACQUITY UPLC i-Class system coupled to a Xevo TQ-S (triple quadrupole MS/MS) Mass Spectrometer (Waters, Manchester, UK). Detection and quantification was achieved using targeted analysis using Multiple Reaction Monitoring (MRM) and operated in ESI^+^ mode. Analyte precursor ions were identified as [M + H]^+^ for both MC-LR and NOD (IS) with the quantifying (*Q*) and qualifying (*q*) ions used as follows:

#### MC-LR

995.6 > 135.03 (*Q*) and 995.6 > 107.05 (*q*)

#### NOD (IS)

825.5 > 135.0*

*Only one transition required due to it being used as an internal standard (IS).

Separation was achieved using an ACQUITY UPLC BEH C18 column, 50 mm × 2.1 mm i.d., 1.8 µm particle size, 130 Å pore size, (Waters, UK) with the column maintained at 45 °C. The mobile phases comprised water (0.1% acetic acid *v/v*) and acetonitrile (ACN) with the flow rate set at 0.45 ml/min. The gradient used to achieve separation consisted of ACN held initially at 2% for 0.5 minute, followed by an increase to 80% over 4 min, washed for 1 min at 90% before returning to 2% for a 1 min re-equilibration time before the next sample injection of 2.5 µL.

#### Analysis of free MC-LR, bound MC-LR (MMPB) and MC-LR-GSH in porcine tissues

For the methodology of the analysis of MC-LR, *free* and *bound*, MC-LR-GSH and NOD (IS), see Supplementary Information.

### Ethics

The animal studies were performed in accordance with a licence issued by the Department of Health, Social Services and Public Safety in NI under the animals (scientific procedures) act 1986 and under the approval of the approval of the Agri-Food and Biosciences Institute (AFBI) Animal Welfare and Ethical Review Body (AWERB), and associated EU Directive 2010/63/EU for animal experiments. The project license is PPL 2755 for chemical contaminants in food producing animals.

## Electronic supplementary material

Supplementary Information
