# Supplementary material for: Uptake and accumulation of Microcystin-LR based on exposure through drinking water: An animal model assessing the human health risk
Source: Sci Rep. 2018 Mar 20;8:4913. doi: 10.1038/s41598-018-23312-7 (PMC5861052; doi:10.1038/s41598-018-23312-7)
Supplement: Supplementary file 1 — Supplementary Information [file 41598_2018_23312_MOESM1_ESM.docx]

**Uptake and accumulation of Microcystin-LR based on exposure through drinking water:**

**An animal model assessing the human health risk**

**Brett Greer^1*^, Julie P. Meneely^1^, Christopher T. Elliott^1^**

^1^Institute for Global Food Security, School of Biological Sciences,

Queens University Belfast, Stranmillis Road, Belfast BT9 5AG, UK

*Corresponding author: brett.greer@qub.ac.uk

**Supplementary Information**

## Standard preparation

MC-LR, NOD internal standard (IS) and MMPB were reconstituted in pure methanol to give stock standards of 1 mg/mL. Working standards of 10 µg/mL were prepared by diluting the stock standards 1:100 v/v with 80% aqueous methanol (v/v). MC-LR and MMPB standards used for method development as well as for preparation of the calibration curves, and NOD (IS) used for spiking into the serum and porcine tissue samples, were prepared at a concentration of 500 ng/mL by dilution of the 10 µg/mL working standards 1:20 (v/v) with 80% aqueous methanol (v/v). To quantify the levels of any MC-LR detected, extracted matrix matched calibration curves were constructed as detailed below. Tissue samples were screened for the depuration product, MC-LR-GSH, however no calibration curve was included for this analyte.

## Method Validation of MC-LR in porcine serum

Toxin extraction and subsequent enrichment was developed using 250 µL aliquots of ‘negative’ porcine serum. Samples were spiked before and after with 50 µL of MC-LR at a concentration of 0.5 µg/mL, equating to a toxin level of 100 ng/mL serum, and 15 µL of the NOD (IS) at 0.5 µg/mL to ascertain extraction efficiency. Once spiked, samples were left for 10 min to allow toxin to disperse through the serum before extraction.

The method was validated to European Decision 2002/657/EC concerning the performance of analytical methods for banned and controlled substances in animals used for food production and was done using 250 µL aliquots of ‘negative’ porcine serum. Validation of the method involved extraction of 20 blank porcine serum samples performed over 3 separate days (seven samples per day), followed by the extraction of 20 spiked porcine serum samples at a concentration of 5 ng/mL (5 ppb) of serum extracted over a further 3 days (seven samples per day). A seven point extracted matrix matched calibration curve was also prepared in the range 0.2 - 100 ng/mL serum. Each sample, including blanks and calibrants were spiked with 15 μL of the NOD (IS) at 0.5 μg/mL. All samples were extracted and analysed as outlined above, giving the inter- and intra-day precision, trueness, absolute recovery, linearity (*R*^2^) and both the CC_α_ (LOD) and CC_β_ (LOQ) values.

## Synthesis of MC-LR-GSH

The depuration product, Microcystin-LR-Glutathione (MC-LR-GSH), was synthesised using the method outlined by Zhang *et al*. (2009) but with minor modifications ^56^. Briefly, MC-LR (1 mg) at a concentration of 10 mg/mL was reacted with 31 mg of L-GSH in a reaction vessel containing 2.5 mL of a 5% aqueous solution of potassium carbonate, stirred at room temperature for 2 h. After the 2 h, the reaction was neutralized by addition of approximately 4 mL of 0.2M hydrochloric acid (HCl).

The reaction mixture was then put through an OASIS HLB SPE cartridge (60 mg, 3 mL) in order to purify the final product. After activation and conditioning of the cartridge with 3 mL of methanol and water respectively, the reaction mixture was loaded onto the cartridge and allowed to drip through under gravity. The cartridge was subsequently washed with 3 mL water and the product eluted into a clean glass vial using 2 x 1.5 mL methanol. Before drying, a 10 µL aliquot of the eluent was removed and transferred to a micro vial for analysis. The remainder was evaporated to dryness using a Turbovap, reconstituted in 1 mL 80% aqueous methanol and stored at -20 °C.

## Determination of MC-LR in porcine samples

### Field Sample Preparation:

***Experiment 1 (dosed at 0.04 µg toxin/kg body weight)***

A sample of porcine serum for each time point from each animal was aliquoted into 2 x 250 µL samples and frozen at -20 °C prior to extraction and analysis. Porcine tissue samples were lyophilised separately using a Christ freeze drier (Osterode, Germany) and subsequently homogenised using a 6850 Freezer/Mill (SPEX SamplePrep LLC, US), after which they were stored at -20 °C prior to extraction and analysis. Four tissues from the eight control and eight treated animals; liver, kidney, small and large intestine were subjected to extraction and analysis, as was serum from every time point for the eight treated animals and pre-bleeds from the eight control and eight treated animals, giving:

Porcine Serum: n=120 (treated), including pre-bleeds from treated and control animals.

Porcine Tissue: n=32 (treated).

***Experiment 2 (dosed at 2 µg toxin/kg body weight)***

Porcine serum and tissues from the twelve animals used in Experiment 2 were prepared and treated exactly as detailed previously and stored at -20 °C prior to extraction and analysis, giving:

Porcine Serum: n=42 (treated) including pre-bleed serum from treated and control animals.

Porcine Tissue: n=36 (treated).

## Synthesis of MC-LR-GSH

Synthesis of MC-LR-GSH, was verified using an ACQUITY UPLC i-Class coupled to a QDa (single quadrupole MS) Mass Spectrometer (Waters, Manchester, UK) operated in electrospray positive mode (ESI^+^). Detection and analysis of MC-LR-GSH was carried out using Selected Ion Recording (SIR). The reactant, MC-LR, was identified as the [M + H]^+^ ion with a mass/charge ratio (m/z) of 995.5, whereas MC-LR-GSH was identified as the [M+2H]^2+^ ion with an m/z of 652.5. Initially, the reactant MC-LR was injected, showing a peak with a retention time (RT) of 3.13 min. Following conjugation, the product was injected and analysed for an m/z of 652.5, showing a peak at RT 2.88 min, verifying a successful synthesis, with both chromatograms shown in Figure S1.

### Field Sample Quantification:

***Analysis of free MC-LR in porcine serum***

To quantify any MC-LR detected, a seven point extracted matrix matched calibration curve in the range 0.2-100 ng/mL serum was prepared using the ‘negative’ porcine serum. These were prepared with the calibrant levels achieved by spiking with the MC-LR standard at either 0.005, 0.05 or 0.5 μg/mL along with spiking each calibrant with 15 μL of the NOD (IS) at 0.5 μg/mL.

***Analysis of free MC-LR in porcine tissues***

In order to quantify any toxins identified, a six-point extracted matrix-matched calibration curve in the range 0.001–0.1 μg/g dw was prepared using pooled control samples of the relevant tissue. These were also prepared as above with the calibrant levels achieved by spiking with the MC-LR standard at either 0.005, 0.05 or 0.5 μg/mL along with spiking each calibrant with 10 μL of the NOD (IS) at 0.5 μg/mL.

### *Analysis of conjugated MC-LR in porcine tissue*

Conversion of any bound MC-LR and subsequent extraction was performed according to a modified method derived from Neffling *et al*. (2010) and developed using 50 mg aliquots of lyophilised homogenised, pooled porcine tissue from the control animals. 50 mg of tissue was weighed into a 50 mL round bottomed flask and 5 mL of oxidation solution at a concentration of 0.1 M was added, comprising both potassium permanganate (KMnO4) and sodium (meta) periodate (NaIO4) at 0.2 M mixed 1:1 *v/v*. The mixture was stirred at room temperature for 2 h whereby the colour of the solution turned from purple (colour of KMnO4) to red/brown (colour of the reduced product MnO2). A further 4 mL oxidation solution at 0.1 M was added turning the solution back to purple. The reaction was immediately quenched by addition of 1 mL sodium bisulphite solution (40% *v/v*). Following this, samples were transferred to 15 mL tubes and 10% sulphuric acid was added drop wise until the pH was acidic (~ 2). Samples were then centrifuged at 4 500 rpm for 15 min and the supernatant removed and loaded directly onto SPE cartridges.

Sample enrichment and clean-up was carried out using OASIS PRiME HLB cartridges, with samples loaded onto the cartridges, allowed to drip through under gravity then washed with water and 20% aqueous methanol (v/v). Bound MMPB was eluted into clean glass tubes using 2 x 1.5 mL 80% aqueous methanol (v/v) and the eluents evaporated to dryness under a gentle stream of nitrogen using a turbovap set at 45 °C. Samples were reconstituted in 100 µL 80% aqueous MeOH (v/v) and transferred to a micro vial for analysis.

**Ultra-performance liquid chromatography tandem mass spectrometry (UPLC- MS/MS)**

### *Analysis of free MC-LR and MC-LR-GSH in porcine tissues*

Analysis of MC-LR, MC-LR-GSH and NOD (IS) was carried out on an ACQUITY UPLC i-Class system coupled to a Xevo TQ-MS (triple quadrupole MS/MS) Mass Spectrometer (Waters, Manchester, UK). Detection and quantification was achieved using MRM and operated in ESI^+^ mode, with MC-LR and NOD (IS) identified as [M + H]^+^ and MC-LR-GSH identified as [M + 2H]^2+^, with the quantifying (*Q*) and qualifying (*q*) ions as follows:

**MC-LR**: 995.6 > 135.03 (*Q*) and 995.6 > 107.05 (*q*)

**NOD (IS)**: 825.5 > 135.0*

**MC-LR-GSH**: 651.95 > 1168.5 (*Q*) and 651.95 > 587.25 (*q*)

*Only one transition required due to it being used as an internal standard (IS).

Separation was achieved using the same column and mobile phases as detailed in ‘*Analysis of free MC-LR in porcine serum’*, however the flow rate was set at 0.4 ml/min. The gradient used to achieve separation consisted of ACN held initially at 5% for 0.5 minute, followed by an increase to 80 % over 4 min, washed for 1 min at 90% before returning to 5% for a 1 min re-equilibration time before the next injection, with this set at 2.5 µL.

### *Analysis of bound MC-LR (MMPB) in porcine tissues*

Analysis of MMPB was performed as described above in ‘*Analysis of free MC-LR and MC-LR-GSH in porcine tissues’.* Detection and quantification was achieved using MRM, with MMPB seen as the [M + H]^+^ ion and the transitions optimised manually as follows:

**MMPB**: 209.15 > 191.2 (*Q*) and 209.15 > 131.1 (*q*)

Separation was achieved using the same column and mobile phases as detailed above in ‘*Analysis of free MC-LR in porcine serum’* with the flow rate set at 0.4 ml/min. The gradient used to achieve separation was as described above with the ACN initially held at 10%, then returned to 10% prior to the next injection.


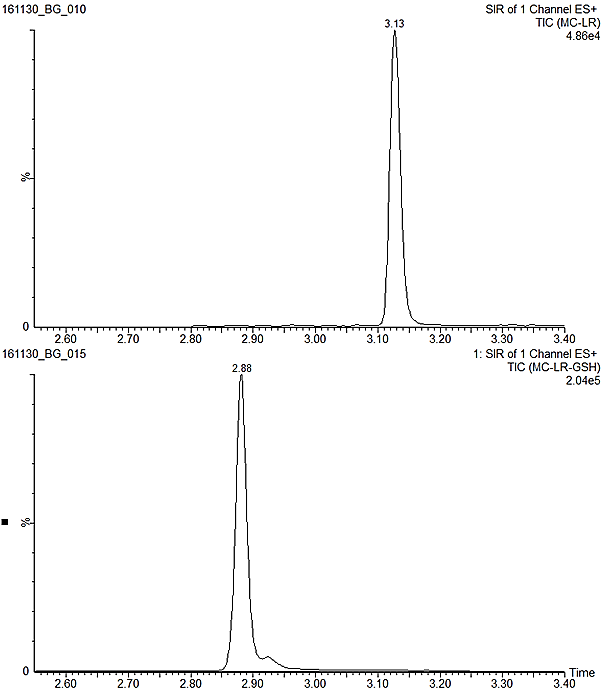


Supplementary Figure S1: Chromatograms from the UPLC-MS (QDa) showing the reactant MC-LR (top) and the product, MC-LR-GSH (bottom), with RTs of 3.13 and 2.88 min respectively.
